# Co-infection of *Fusarium aglaonematis* sp. nov. and *Fusarium elaeidis* Causing Stem Rot in *Aglaonema modestum* in China

**DOI:** 10.3389/fmicb.2022.930790

**Published:** 2022-06-30

**Authors:** Yunxia Zhang, Chao Chen, Zhanglong Mai, Jieying Lin, Liting Nie, Sajeewa S. N. Maharachchikumbura, Chunping You, Meimei Xiang, Kevin D. Hyde, Ishara S. Manawasinghe

**Affiliations:** ^1^Innovative Institute for Plant Health, Zhongkai University of Agriculture and Engineering, Guangzhou, China; ^2^Key Laboratory of Green Prevention and Control on Fruits and Vegetables in South China, Ministry of Agriculture and Rural Affairs, Zhongkai University of Agriculture and Engineering, Guangzhou, China; ^3^School of Life Science and Technology, Center for Informational Biology, University of Electronic Science and Technology, Chengdu, China; ^4^Center of Excellence in Fungal Research, Mae Fah Luang University, Mueang, Chiang Rai, Thailand

**Keywords:** new species, co-infection, pathogenicity, *Nectriaceae*, Sordariomycetes

## Abstract

*Aglaonema modestum* (*A. modestum*) (Araceae) is an evergreen herbage, which is intensively grown as an ornamental plant in South China. A new disease was observed in *A. modestum* from 2020 to 2021 in Guangdong province, China. The disease symptoms associated with plants were initial leaf wilt, stem rot, and resulting plant death, leading to severe economic losses. In total, six *Fusarium* isolates were obtained from diseased plants. The putative pathogen was identified using both morphological characteristics and molecular phylogenetic analysis of calmodulin A (*cmdA*), RNA polymerase largest subunit 1 (*rpb1*), RNA polymerase II (*rpb2*), translation elongation factor-1α (*tef1-*α), and beta-tubulin (β*-tubulin*) sequences. Two *Fusarium* species were identified, namely, one new species, *Fusarium aglaonematis* (*F. aglaonematis*) belonging to *Fusarium fujikuroi* species complex. In addition, *Fusarium elaeidis* (*F. elaeidis*) belonging to the *Fusarium oxysporum* (*F. oxysporum*) species complex was also identified. Pathogenicity assays were conducted by inoculating each species into potted *A. modestum* plants and co-inoculating two species. The results showed that two *Fusarium* species could infect plants independently and can infect them together. Co-infection of these two species enhanced the disease severity of *A. modestum*. Compared to single inoculation of *F. elaeidis*, severity was higher and disease development was quicker when plants were only inoculated with *F. aglaonematis*. In addition, these two *Fusarium* species could infect *Aglaonema* plants without wounds, while inoculation with a physical injury increased disease severity. This is the first report of co-infection by *F. aglaonematis* and *F. elaeidis* causing stem rot on *A. modestum* worldwide. This study will be an addition to the knowledge of *Fusarium* diseases in ornamental plants. These results will provide a baseline to identify and control diseases associated with *A. modestum.*

## Introduction

Ornamental plants are an important commodity in China due to their aesthetic value and economic importance. Guangdong is one of the largest ornamental plant cultivating regions in China (Han, 2014). Ornamental plant production accounts for an important position in the economic development of Guangdong province. Therefore, to promote the economic impact of this industry, commercialization of plant production and large-scale cultivation are facilitated. With the rapid development of the ornamental plant industry, diseases have become an emerging problem that affects plant production. It is necessary to identify pathogens for early detection and control, especially in commercial nurseries where several plant generations will be established within a controlled environment (Jayawardena et al., 2021; Manawasinghe et al., 2021).

Moreover, it is also important to understand the interactions between different pathogens. Disease complex usually involved multipathogen interactions, such as fungal–fungal, fungal–bacteria, fungal–nematode, closely related species, and so on. For example, both Botryosphaeriaceae and *Ilyonectria* spp. contributed to the decline of young grafted grapevines (Whitelaw-Weckert et al., 2013), the combination of *Didymella bryoniae* and pathogenic bacteria resulted in disease on Styrian oil pumpkin (Grube et al., 2011), co-infection of *Fusarium oxysporum* (*F. oxysporum*) f. sp. *lycopersici* and nematode (*Meloidogyne* species) on tomato caused serious damage in Ethiopia (Kassie, 2019), and co-occurrence of two strains of the cassava mosaic virus led to severe symptoms on leaves of *Nicotiana benthamiana* (Fondong et al., 2000). Due to multipathogen interactions (competition, cooperation, or coexistence), co-infections have complex mechanisms compared to single-pathogen induced systems (Abdullah et al., 2017). Thus, understanding the pathogenicity mechanisms with co-infections is necessary.

*Aglaonema* belongs to the Araceae (Juss.), which comprises 50 plant species and is one of the commercial scaled ornamental plants in China. Only two species, *Aglaonema modestum* (*A. modestum*) and *A. tenuipes* are grown in China^1^. *Aglaonema modestum*, also called ‘‘Chinese evergreen,’’ is a flowering plant native to Bangladesh, Thailand, Laos, Vietnam, and southeast and south-central China^2^. *Aglaonema modestum* is commercially grown in Guangdong province as a foliage ornamental plant because of its aesthetic and economic value. Until today, few diseases have been reported on *Aglaonema* plants, namely, bacterial leaf blight, viral and nematode diseases, and four fungal diseases (Ann, 1992; Marban and Flores, 1993; Uchida and Aragaki, 1994; Fawzy, 1996; Gupta and Sunita, 1996; Chao et al., 2006; Mounika et al., 2017). *Colletotrichum gloeosporioides* has been reported to cause anthracnose on *Aglaonema crispum*, which led to devastating foliar damage (Mounika et al., 2017). *Phytophthora* blight caused by *P. meadii* and *P. nicotianae* var. *parasitica* on *A. nitidum* and *A. commutatum* (Ann, 1992) has been reported from Taiwan, China. Collar rot and foliar blight disease caused by *F. subglutinans* have been reported from Hawaii, United States (Uchida and Aragaki, 1994). *Sphaeropsis modestum* has been reported to cause leaf blight in India (Gupta and Sunita, 1996). However, little is known about diseases caused by common fungal genera such as *Fusarium* in China.

*Fusarium* is one of the well-known phytopathogenic genera that belong to the *Nectriaceae* (Hyde et al., 2020). *Fusarium* accommodates 18 species complexes (Laurence et al., 2011; O’Donnell et al., 2013; Aoki et al., 2014; Zhou et al., 2016; Sandoval-Denis et al., 2018; Lombard et al., 2019; Crous et al., 2021) with over 100 species. These species represent some of the most devastating plant pathogens worldwide (Wang et al., 2022). In recent years, many *Fusarium* diseases have been reported to be associated with ornamental plants (Gullino et al., 2012, 2015; Srivastava et al., 2018; Muaz et al., 2020; He et al., 2021; Kamali-Sarvestani et al., 2022). Srivastava et al. (2018) showed at least eight *Fusarium* species associated with orchid diseases, namely, leaf spots, sheath blights, pseudostem or root rots, and wilts. *Fusarium oxysporum* and *F. proliferatum* were recovered from more than 27 diverse *Cactus* species and other succulent plants causing *Fusarium* dry rot and soft rot (Kamali-Sarvestani et al., 2022). *Fusarium incarnatum* was reported to infect *Gerbera jamesonii* and causes stem and root rot (Chen et al., 2021). Moreover, in China, *F. rosicola* was identified as a causal agent of vascular wilt on *Rosa chinensis* (He et al., 2021). Thus, it is necessary to identify and characterize *Fusarium* species associated with ornamental plant species, especially in large-scale commercial cultivations.

During 2020–2021, a new severe disease was observed in commercially cultivated *A. modestum* plants in Guangzhou city, Guangdong province, China. Approximately, 60% of the 50,000 plants with stem rot were discovered, leading to significant economic losses. The objectives of this study were to: (i) isolate and identify the causal agents of this disease and (ii) understand the possibility of co-infection among isolated taxa. The pathogens were identified based on both morphological characteristics and phylogenetic analyses. The pathogenicity assays were conducted on potted *A*. *modestum* plants, and Koch’s postulates were fulfilled.

## Materials and Methods

### Sample Collection

From 2020 to 2021, typical wilting symptoms were observed in approximately 60% of the 50,000 plants at a commercial nursery in Guangdong province, China. The symptoms were as follows: plant collars were completely rotted and leaves became yellow, wilted, and frequently collapsed. A total of 20 disease plants with typical symptoms were collected. Disease symptoms were recorded and relevant pictures were taken. Then, the samples were taken into the laboratory using Ziplock bags for further studies.

### Fungal Isolation and Purification

Infected stems from diseased plants were washed with running tap water to remove debris. Pathogen(s) were isolated by the tissue isolation method (Senanayake et al., 2020; Zhang et al., 2021). Diseased tissues were cut into small pieces (0.5 cm × 0.5 cm) taken from the margin of infected stems and healthy tissues. The cuttings were immersed in 75% ethanol for 15 s, 2.5% NaClO for 15 s, and rinsed in sterile distilled water three times. Tissue pieces were blotted dry in sterile paper towels and incubated on potato dextrose agar (PDA) at 25°C. Pure cultures were obtained after single spore isolation. In total, six isolates were obtained from diseased stems. All the cultures obtained in this study are deposited in the culture collection of Zhongkai University of Agriculture and Engineering (ZHKU). Herbarium materials (as dry cultures) are deposited at Zhongkai University of Agriculture and Engineering (ZHKU).

### Morphological Characterization

Colony morphology and pigmentation were documented on PDA after 5 and 7 days at 25°C with a 12-h light-dark cycle. Colony growth rates were determined on PDA by inoculating overgrown 6-mm agar blocks. Morphological characters were observed after pure culture was grown on carnation leaf agar (CLA; Fisher et al., 1982), incubated at 25°C under a 12-h light-dark cycle. Morphological characters were photographed using an ECLIPSE 80i microscope (Nikon, Tokyo, Japan) and measurements were taken using NIS-Elements BR 3.2. Fifty measurements were made for the conidia and other morphological structures. The mean values were calculated with Microsoft Excel.

### Deoxyribonucleic Acid Extraction, Polymerase Chain Reaction Amplification, and Sequencing

Based on colony morphology and morphological characters, we identified our isolates belong to *Fusarium* (Wang et al., 2022). The genomic DNA was extracted using the DNA Rapid Extraction Kit (Aidlab Biotechnologies Corporation Ltd., Beijing, China) from 5-day-old cultures grown on PDA. For the molecular characterization of six isolates, five gene regions, namely, partial sequences of calmodulin A (*cmdA*), RNA polymerase largest subunit 1 (*rpb1*), RNA polymerase second largest subunit (*rpb2*), translation elongation factor 1-alpha (*tef1-*α), and beta-tubulin (β*-tubulin*) were employed. The polymerase chain reaction (PCR) reaction mixture of 25 μl consisted of 12.5 μl of 2X Easy Taq PCR SuperMix (TransGen Biotech, Beijing, China), 1 μl DNA, each of 5 μM premier (1 μl), and ddH_2_O (9.5 μl). For each gene region, primer pairs and respective PCR amplification protocols are given in Table 1. The PCR products were sequenced by Guangzhou Tianyi Huiyuan Science and Technology Corporation Ltd. (Guangzhou, China). Both directions were sequenced using forward and reverse primers for each gene region. Sequencing quality was assured by checking sequence chromatograms using BioEdit v.7.0.5.2 (Hall, 1999). The combined sequences were generated using forward and reverse primers using BioEdit v.7.0.5.2 (Hall, 1999). Newly generated sequences in this study are deposited in the GenBank (Supplementary Tables 1, 2).

**TABLE 1:** Gene regions and respective primer pairs used in the study.

Locus	Primer	Sequence (5′-3′)*	PCR amplification on procedures	References
*cmdA*	CL1	GARTWCAAGGAGGCCTTCTC	94°C 90 s; 35 cycles of 94°C 45 s, 50°C 45 s, 72°C 1 min; 72°C 10 min; 16°C soak	O’Donnell et al. (2000)
	CL 2A	TTTTTGCATCATGAGTTGGAC		
*tef1-*α	EF1H	ATGGGTAAGGAAGACAAGAC	95°C 3 min; 35 cycles of 94°C 30 s, 56°C 45 s, 72°C 1 min; 72°C 10 min; 16°C soak	O’Donnell (2000)
	EF2T	GGAAGTACCAGTGATCATGTT		
	EF1	ATGGGTAAGGARGACAAGAC	95°C 3 min; 35 cycles of 95°C 30 s, 52°C 30 s, 72°C 1 min; 72°C 10 min; 16°C soak	O’Donnell et al. (1998)
	EF2	GGARGTACCAGTSATCATG		
β*-tubulin*	T1	AACATGCGTGAGATTGTAAGT	95°C 3 min; 35 cycles of 95°C 30 s, 53°C 30 s; 72°C 1 min; 72°C 10 min; 16°C soak	O’Donnell and Cigelnik (1997)
	CYLTUB1R	AGTTGTCGGGACGGAAGAG		Crous et al. (2004)
	T1	AACATGCGTGAGATTGTAAGT	95°C 3 min; 35 cycles of 95°C 30 s, 52°C 30 s, 72°C 1 min; 72°C 10 min; 16°C soak	O’Donnell and Cigelnik (1997)
	T2	TAGTGACCCTTGGCCCAGTTG		
*rpb1*	Fa	CAYAARGARTCYATGATGGGWC	94°C 90 s; 5 cycles of 94°C 45 s, 54°C 45 s, 72°C 2 min; 5 cycles of 94°C 45 s, 53°C 45 s, 72°C 2 min; 35 cycles of 94°C 45 s, 52°C 45 s, 72°C 2 min; 72°C 10 min; 10°C soak	Hofstetter et al. (2007)
	R8	CAATGAGACCTTCTCGACCAGC		O’Donnell et al. (2010)
	F8	TTCTTCCACGCCATGGCTGGTCG	94°C 90 s; 5 cycles of 94°C 45 s, 56°C 45 s, 72°C 2 min; 5 cycles of 94°C 45 s, 55°C 45 s, 72°C 2 min; 35 cycles of 94°C 45 s, 54°C 45 s, 72°C 2 min; 72°C 10 min; 10°C soak	O’Donnell et al. (2010)
	G2R	GTCATYTGDGTDGCDGGYTCDCC		O’Donnell et al. (2010)
*rpb2*	5f	GAYGAYMGWGATCAYTTYGG	95°C 5 min; 35 cycles of 94°C 1 min, 53°C 30 s, 72°C 90 s; 72°C 10 min; 16°C soak	Liu et al. (1999)
	7cr	CCCATRGCTTGYTTRCCCAT		
	5F2	GGGGWGAYCAGAAGAAGGC	95°C 5 min; 35 cycles of 94°C 1 min, 52°C 30 s, 72°C 90 s; 72°C 5 min; 16°C soak	Reeb et al. (2004)
	7Cr	CCCATRGCTTGYTTRCCCAT		Liu et al. (1999)
	7Cf	ATGGGYAARCAAGCYATGGG	95°C 5 min; 35 cycles of 94°C 1 min, 52°C 30 s, 72°C 90 s; 72°C 5 min; 16°C soak	Liu et al. (1999)
	11ar	GCRTGGATCTTRTCRTCSACC		

*Y = T or C; M = A or C; W = A or T; R = A or G.

### Phylogenetic Analyses

To confirm the *Fusarium* species complex, *tef1-*α sequences were subjected to the Basic Local Alignment Search Tool (BLASTn)^3^ at the National Center for Biotechnology Information (NCBI). We identified our isolates belonging to *Fusarium fujikuroi* (*F. fujikuroi*) species complex (FFSC) and *F. oxysporum* species complex (FOSC). Sequences of *F. fujikuroi* species complex (FFSC) were downloaded from the NCBI following a study by Crous et al. (2021); Yilmaz et al. (2021), and Wang et al. (2022; Supplementary Table 1). Sequences of *F. oxysporum* species complex (FOSC) were downloaded from the NCBI following a study by Lombard et al. (2019; Supplementary Table 2). Downloaded sequences were aligned together with sequences from this study using MAFFT v.7^4^ (Katoh and Toh, 2010). Using BioEdit 7.0.5.2, sequences were improved manually when necessary (Hall, 1999). Combined sequences dataset of five loci, namely, *cmdA*, *rpb1*, *rpb2*, *tef1-*α, and β*-tubulin*, were used for phylogenetic analyses of FFSC following a study by Crous et al. (2021); Yilmaz et al. (2021), and Wang et al. (2022). Combined sequences dataset of three loci, namely, *rpb2*, *tef1-*α, and β*-tubulin*, were used for phylogenetic analyses of FOSC following a study by Lombard et al. (2019).

In the present study, phylogenetic analyses were inferred using maximum likelihood (ML) in RAxML (Silvestro and Michalak, 2012) and Bayesian posterior probability (BYPP) analysis in MrBayes (v3.0b4) (Ronquist and Huelsenbeck, 2003). Maximum likelihood analyses were carried out using RAxML-HPC2 on XSEDE (8.2.8) on the CIPRES science gateway platform^5^ (Miller et al., 2010). The GTR + I + G evolution model was used with 1,000 non-parametric bootstrapping iterations. Bayesian analysis was performed with six simultaneous Markov chains run for 10^6^ generations, sampling the trees at every 200th generation. From the 10,000 trees obtained, the first 2,000 trees representing the burn-in phase were discarded. The remaining 8,000 trees were used to calculate posterior probabilities (PPs) in a majority rule consensus tree. The stability of the trees was evaluated by 1,000 bootstrap replications. Descriptive statistics were calculated for the resulting tree. Phylogenetic trees were visualized in FigTree v.1.4.2. Taxonomic novelties were submitted to the Index Fungorum^6^.

### Pathogenicity Tests and Co-inoculation

Three representative isolates for each *Fusarium* species were used to inoculate potted *A. modestum* plants. Representative isolates grown on PDA at 25°C for 4 days were used to make 1 × 10^5^ spores/ml spore suspension. *Aglaonema modestum* plants with and without physical wounds (cutoff three fibrous roots from each stem using a sterilized blade) were inoculated as follows: A; inoculating *Fusarium aglaonematis* (*F. aglaonematis*) alone (ZHKUCC 220077, ZHKUCC 220078, and ZHKUCC 220079), B; inoculating *Fusarium elaeidis* (*F. elaeidis*) alone (ZHKUCC 220080, ZHKUCC 220081, and ZHKUCC 220082), and C; co-inoculation of two *Fusarium* species combined as ZHKUCC 220077 + ZHKUCC 220080, ZHKUCC 220078 + ZHKUCC 220081, and ZHKUCC 220079 + ZHKUCC 220082. In total, 150 ml of spore suspension was used to inoculate each pot. For each treatment (in a total of nine treatments), three replicates were used and sterilized water was used as the control. Inoculated plants were maintained at 25°C in a growth chamber. To fulfill Koch’s postulates, fungi were reisolated from inoculated plants with typical symptoms and identified morphologically. Disease severity was estimated and rated as follows: 0 = no disease, 1 = small lesions with under 25% of stem internal affected, inoculation plants did not lodge; and 2 = large lesions over 25% of stem internal affected, inoculation plants lodged. Disease index (DI) was calculated as follows: DI = [Σ (numbers of diseased plants of each rate × rate number)/(total numbers of inoculation plants × highest rate number)] × 100.

## Results

### Disease Symptoms and Isolation

**FIGURE 1:**
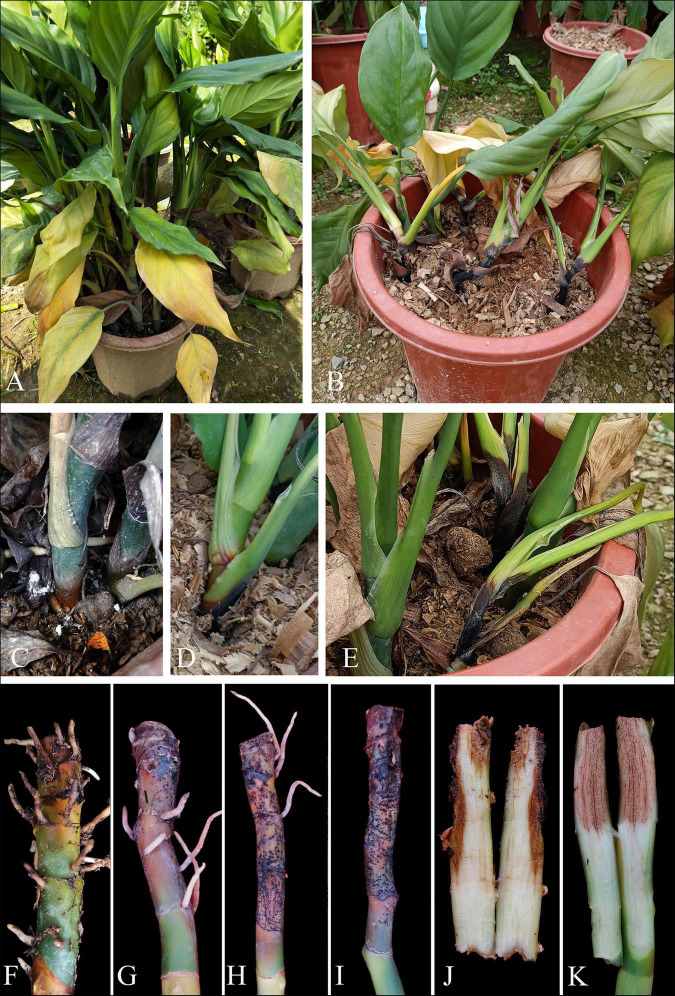
Field symptoms of stem root rot on *Aglaonema modestum*. **(A,B)** Symptoms of whole plants. **(C–E)** Stem rot on potted plants. **(F–I)** Progressive stem rot. **(J,K)** Internal symptoms of stem rot.

This disease began with gradual leaf wilt and sometimes a progressive yellowing of the foliage, and finally, whole plants collapsed (Figures 1A,B). Initial symptoms on stems appeared as water-soaked spots. These spots appeared around the root, then expanded to a large brown spot, followed by depressed black necrotic lesions (Figures 1C–I). Then, diseased plants were lodged (Figure 1B). Internally, diseased stems are brown and spongy (Figures 1J,K). When the pathogen was isolated from diseased field samples, *F. elaeidis* was the most dominant. For *F. elaeidis*, 26 colonies were grown from 26 tissue pieces out of 35 tissue pieces used. For *F. aglaonematis*, only five colonies were grown from five tissue pieces out of 35 tissue pieces. However, on-field plants, only *F. aglaonematis* was observed on rotted stems, on which it produced fungal structures without sporulation.

### Phylogenetic Analyses

**FIGURE 2:**
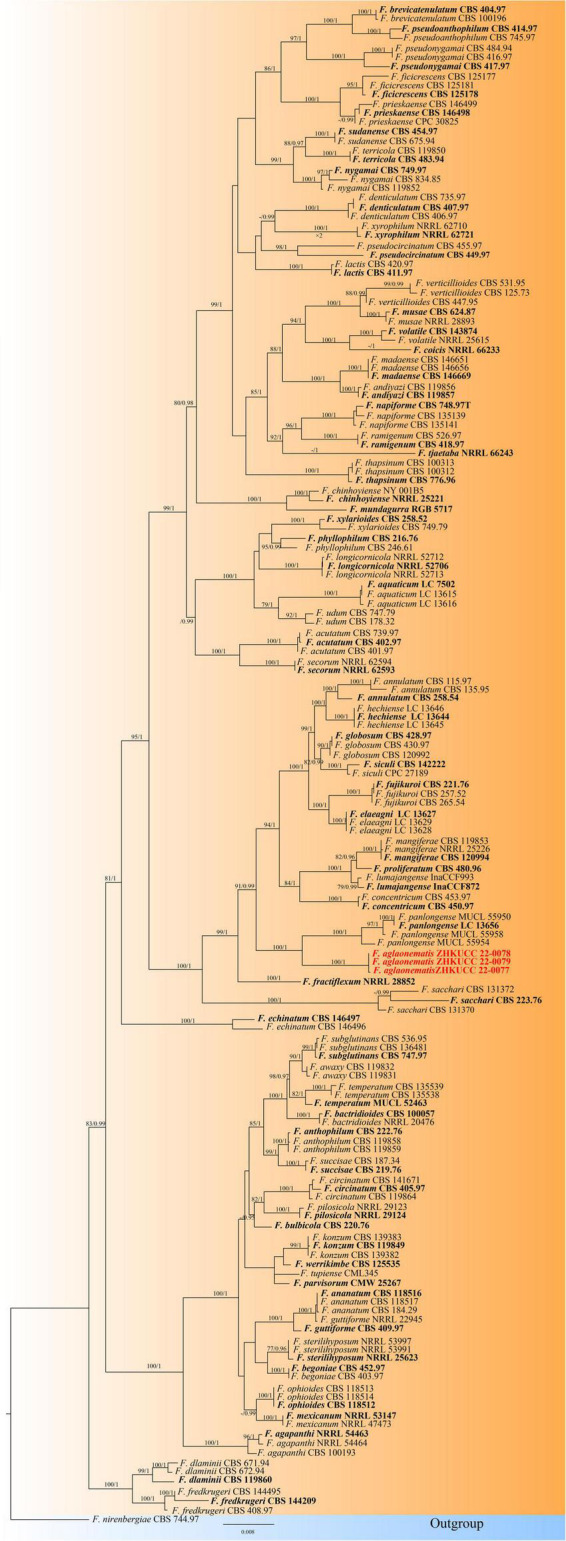
The Best scoring RAxML tree obtained from the combined *cmdA*, *rpb1*, *rpb2*, *tef1-α*, and *β-tubulin* sequence alignment of the *Fusarium fujikuroi* complex. The maximum likelihood (ML) bootstrap support values ≥75% and Bayesian posterior probability (BYPP) higher than 0.90 are indicated at the nodes and branches. The scale bar indicates 0.07 changes per site. Ex–type/ex–epitype strains are in bold. New isolates recovered in this study are in red. *Fusarium nirenbergiae* (CBS 744.97) was used as an outgroup.

In the present study, two phylogenetic trees were generated for *F. fujikuroi* species complex and *F. oxysporum* species complex. The phylogenetic tree of *Fusarium fujikuroi* species complex was generated using the combined data set of *cmdA*, *rpb1*, *rpb2*, *tef1-*α, and β*-tubulin* sequence data. In total, 161 *Fusarium* strains were used, including three strains from the present study. *Fusarium nirenbergiae* (CBS 744.97) was used as the outgroup. The tree topology of ML analysis was similar to the BYPP analysis. The best-scoring RAxML tree with a final likelihood value of −28242.724276 is presented (Figure 2). The matrix had 1,604 distinct alignment patterns, with 19.12% of undetermined characters or gaps. Estimated base frequencies were as follows: *A* = 0.255170, *C* = 0.253256, *G* = 0.246235, *T* = 0.245339; substitution rates AC = 1.316349, AG = 6.033197, AT = 1.215071, CG = 0.920367, CT = 11.198152, GT = 1.000000; and gamma distribution shape parameter α = 0.772643. Three isolates from this study formed a distinct clade with 100% ML bootstrap support and 1.00 BYPP with close relation to *Fusarium panlongense* (*F. panlongense*).

**FIGURE 3:**
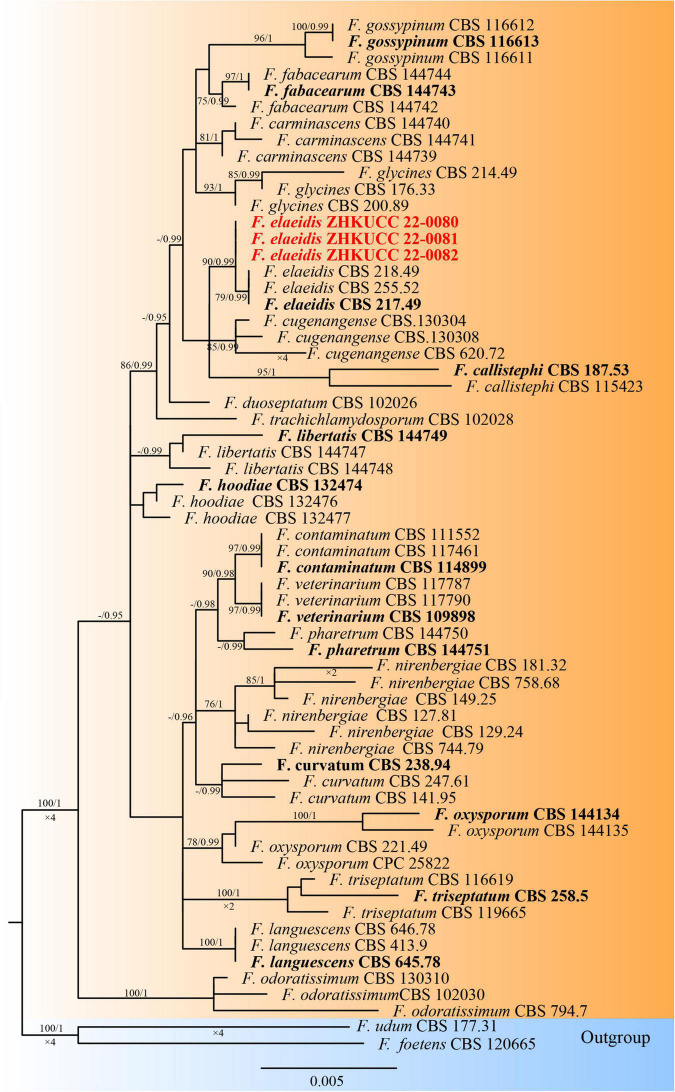
The Best scoring RAxML tree obtained from the combined with *rpb2*, *tef1-α*, and *β-tubulin* sequence alignment of the *Fusarium oxysporum* complex. The ML bootstrap support values ≥75% and BYPP higher than 0.90 are indicated at the nodes and branches. The scale bar indicates 0.005 changes per site. Ex–type/ex–epitype strains are in bold. New isolates recovered in this study are in red.

The phylogenetic tree of the *Fusarium oxysporum* species complex was generated using combined *rpb2*, *tef1-*α, and β*-tubulin* sequence data. Sixty-three *Fusarium* strains were used, including three isolates from the present study. *Fusarium udum* (CBS 177.31) and *Fusarium foetens* (CBS 120665) were used as the outgroup taxa. The tree topology of the ML analysis was similar to the BYPP. The best-scoring RAxML tree with a final likelihood value of −5610.450841 is presented (Figure 3). The matrix had 258 distinct alignment patterns, with 0.99% of undetermined characters or gaps. Estimated base frequencies were as follows: *A* = 0.251049, *C* = 0.269367, *G* = 0.238927, and *T* = 0.240658; substitution rates AC = 1.318036, AG = 3.197313, AT = 0.814686, CG = 0.935161, CT = 6.472797, and GT = 1.000000; and gamma distribution shape parameter α = 1.036370. Three isolates from this study clustered with *F. elaeidis* with 90% ML bootstrap support and 0.99 BYPP, separately from other species.

### Taxonomy

**FIGURE 4:**
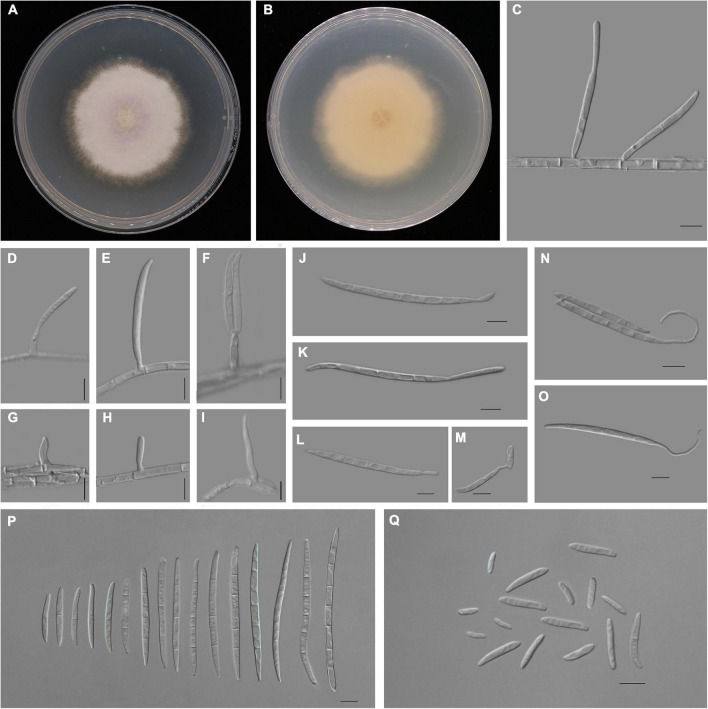
*Fusarium aglaonematis* (ZHKUCC 22-0077). **(A,B)** Culture characteristics on potato dextrose agar (PDA) after 7 days [**(A)** upper view; **(B)** reverse view]. **(C)** Macroconidia with up-dip attachment from mycelium. **(D–F)** Phialides with aerial macroconidia. **(G–I)** Phialides of aerial microconidia. **(J–M)** Macroconidia with up-dip attachment. **(N,O)** Macroconidia germination. **(P)** Aerial macroconidia. **(Q)** Aerial microconidia. Scale bars: **(C–Q)** = 10 μm.

*Fusarium aglaonematis* Y. X. Zhang, C. Chen and Manawas, sp. nov. (Figure 4).

Index Fungorum number: IF555872.

Etymology—Epithet refers to the plant from which the type was collected.

Holotype—ZHKUCC 22-0077.

*Pathogenic* on *A. modestum*. *Sexual morphology*: Not observed. *Asexual morphology*: *Sporodochia* is not observed. *Aerial macroconidia* straight to falcate, tapering toward the basal part, robust, moderately curved and slender, apical cell papillate; basal cell foot-shaped to barely notched, (0–)1–3(–5)-septate; 0-septate *conidia*: 25–35 μm × 2–3 μm (x̄ = 32 μm × 3 μm, *n* = 8); 1-septate conidia: 20–30 μm × 3–4 μm (x̄ = 25 μm × 3 μm, *n* = 50); 2-septate conidia: 40–55(–65) μm × 3–4 μm (x̄ = 49 μm × 4 μm, *n* = 9); 3-septate conidia: (25–)45–80 μm × 3–5 μm (x̄ = 61 μm × 4 μm, *n* = 50); 4-septate conidia: 60–75(–80) μm × 3–4 (–5) μm (x̄ = 68 μm × 4 μm, *n* = 6); and 5-septate conidia: 85–95 μm × 4–5 μm (x̄ = 90 μm × 4 μm, *n* = 2). Some macroconidia with up dip attachment. *Aerial microconidia* forming small false heads on the tips of the phialides, *conidiogenous cells* 5–20(–30) μm × (2–)3(–4) μm (x̄ = 14 μm × 3 μm, *n* = 50); *microconidia* reniform to subclavate, smooth- and thin-walled, 0–1(–2)-septate; 0-septate conidia: 5–25 μm × 2–5 μm (x̄ = 15 μm × 3 μm, *n* = 50); 1-septate conidia: 20–30 μm × 3–4 μm (x̄ = 25 μm × 3 μm, *n* = 22); *Chlamydospores* are not observed.

Culture characteristics: Colony growth rate was 3.3 mm on PDA at 25°C per day. The colony surface floccose with a regular margin, white at first and turned light purple in the center at the end. In the reverse colony, it is white.

Material examined: China, Guangdong province, Guangzhou city, *Aglaonema modestum* Schott ex Engl., (*Araceae*). 23 August 2020, YX Zhang (ZHKU 22-0044, holotype); dry cultures ZHKU 22-0045, ZHKU 22-0046 paratype, and living culture ZHKUCC 22-0077 ex–holotype; ZHKUCC 22-0078, ZHKUCC 22-0079 ex–paratype.

Notes: In the phylogenetic tree, three isolates in this study formed a lineage with 100% ML bootstrap support and a 1.00 BYPP value. Morphologically, *F. aglaonematis* is distinguished from its closely related taxa, *F. panlongense* by the size of microconidia and macroconidia (Wang et al., 2022; Table 2). The length of macroconidia and microconidia of new species is longer than those of *F. panlongense* described in a study by Wang et al. (2022; Table 2). In addition, *F. aglaonematis* grows slowly (46 mm diameter on PDA after 7 days at 25°C) than *F. panlongense* (57–59 mm diameter on PDA after 7 days at 25°C). When five gene regions were compared (without gaps) between *F. aglaonematis* (ZHKUCC 22-0044) and *F. panlongense* (LC13656), 5 base pairs difference in *cmdA* (totally 610 bp), 18 base pairs difference in *rpb1* (totally 1,525 bp), 48 base pairs difference in *rpb2* (totally 1,563 bp), 27 base pairs difference in *tef1-*α (totally 613 bp), and 1 base pair difference in β*-tubulin* (totally 471 bp). Based on phylogenetic analyses and morphology, the isolates causing *A. modestum* stem rot were identified as a new species.

**TABLE 2:** Comparison of morphological characteristics between *Fusarium aglaonematis* and *F. panlongense.*

Species	Microconidia (μm)		Macroconidia (μm)					References
	0-septate	1-septate	1-septate	2-septate	3-septate	4-septate	5-septate	
*F. panlongense*	5–8 × 1.5–3 x̄ = 6 × 2	8–14 × 2–3.5 x̄ = 11 × 3	N/A	N/A	37–50 × 3–4 x̄ = 42 × 4	40–53 × 2.5–6 x̄ = 48 × 4	46–58 × 2.6–5 x̄ = 51 × 4	Wang et al., 2022
*F. aglaonematis*	5–25 × 2–5 x̄ = 15 × 3	20–30 × 3–4 x̄ = 25 × 3	20–30 × 3–4 x̄ = 25 × 3	40–55 × 3–4 x̄ = 49 × 4	45–80 × 3–5 x̄ = 61 × 4	60–75 × 3–4 x̄ = 68 × 4	85–95 × 4–5 x̄ = 90 × 4	This study

**FIGURE 5:**
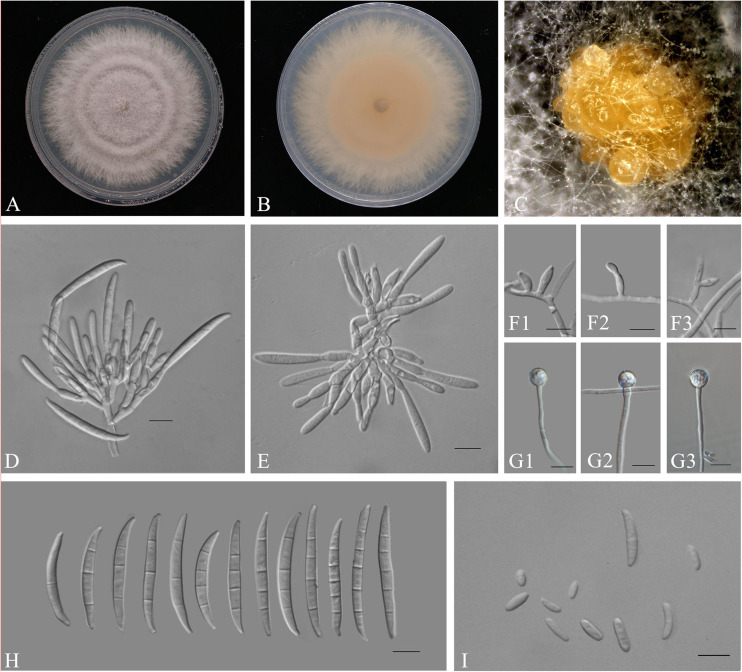
*Fusarium elaeidis* (ZHKUCC 22-0080). **(A,B)** Culture characteristics on PDA after 7 days [**(A)** upper view; **(B)** reverse view]. **(C)** Sporodochia formed on the surface of carnation leaves. **(D,E)** Sporodochial conidiophores and phialides. **(F1–F3)** Aerial conidiophores and phialides. **(G1–G3)** Chlamydospores. **(H)** Sporodochial conidia. **(I)** Aerial conidia. Scale bars: **(D–I)** = 10 μm.

***Fusarium elaeidis*** L. Lombard and Crous, in Lombard, Sandoval-Denis, Lamprecht and Crous, Persoonia 41:23 (2018) (Figure 5).

Index Fungorum number: IF 826838.

Pathogenic on *A. modestum*. *Sexual morphology*: Not observed. *Asexual morphology*: *Sporodochia* pale orange, formed abundantly on carnation leaves. *Sporodochial conidiogenous cells* 7–20 μm × 2–4 μm (x̄ = 12 μm × 3 μm *n* = 50), doliiform to subcylindrical, smooth and thin-walled, with periclinal thickening and an inconspicuous apical collarette. *Sporodochial macroconidia* falcate, curved dorsoventrally with almost parallel sides tapering slightly toward both ends, with a blunt to papillate, curved apical cell, and a blunt to foot-like basal cell, 3–5-septate, hyaline, smooth- and thin-walled; 3-septate conidia: 30–50 μm × 3–5(–6) μm (x̄ = 38 μm × 4 μm, *n* = 50); 4-septate conidia: 35–55(–60) μm × 3–5(–6) μm (x̄ = 45 μm × 4 μm, *n* = 50); and 5-septate conidia: 40–50(–55) μm × 4–5 μm (x̄ = 47 μm × 4 μm, *n* = 15). *Conidiogenous cells* 5–15 μm × 2–4 μm (x̄ = 9 μm × 3 μm, *n* = 50), subulate, to subcylindrical, smooth- and thin-walled; aerial microconidia forming small false heads on the tips of the phialides, 0–1-septate; 0-septate conidia: 5–15(–20) μm × 2–3 μm (x̄ = 8 μm × 3 μm, *n* = 50); 1-septate conidia: 10–25 μm × 2–4 μm (x̄ = 18 μm × 3 μm, *n* = 16). *Chlamydospores* 5–10 μm × 5–10 μm (x̄ = 9 μm × 8 μm), formed solitary, in pairs or chains, either terminal or intercalary in hyphae.

Culture characteristics: Colony growth rate was 5.1 mm on PDA at 25°C per day. The colony surface was light purple, flat, and floccose with a regular margin. In the reverse colony, it is white at first and turned rosy vinaceous in the center at the end; on CLA, aerial mycelium is sparse with abundant orange sporodochia forming on the carnation leaves.

Material examined: China, Guangdong province, Guangzhou city, *Aglaonema modestum* Schott ex Engl., (*Araceae*). 31 March 2021, YX Zhang (dried cultures ZHKU 22-0047, ZHKU 22-0048, and ZHKU 22-0049; living cultures, ZHKUCC 22-0080, ZHKUCC 22-0081, and ZHKUCC 22-0082).

Notes: In the phylogenetic tree, three isolates in this study clustered with *Fusarium elaeidis* with 90% ML and 0.99 BYPP. Morphologically, the microconidia length of our isolates is longer than those of microconidia as described in a study by Lombard et al. (2019). Therefore, based on phylogenetic analyses and morphological analyses, our isolates from *A. modestum* were identified as *F. elaeidis.*

### Pathogenicity Test and Co-infection

**FIGURE 6:**
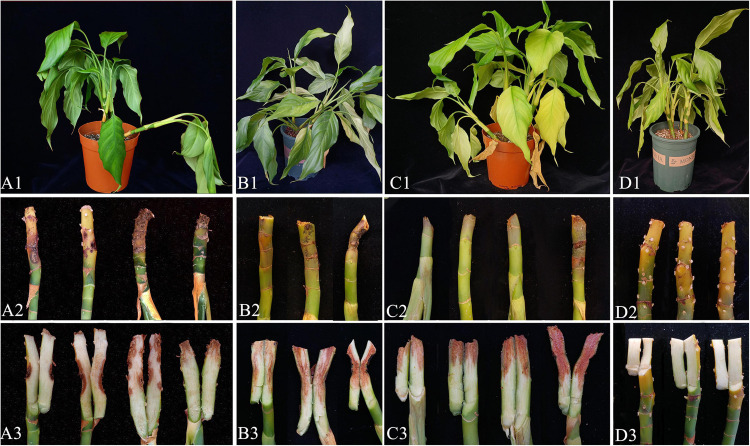
Inoculation test results of *Alocasia modestum* stem rot (*A. modestum*). **(A1–A3)** Inoculation with *F. elaeidis* only. **(B1–B3)** Inoculation with *F. aglaonematis* only. **(C1–C3)** Inoculation with both *F. elaeidis* and *F. aglaonematis.* **(D1–D3)** Control plants of *A. modestum* after 15 days of inoculation.

**FIGURE 7:**
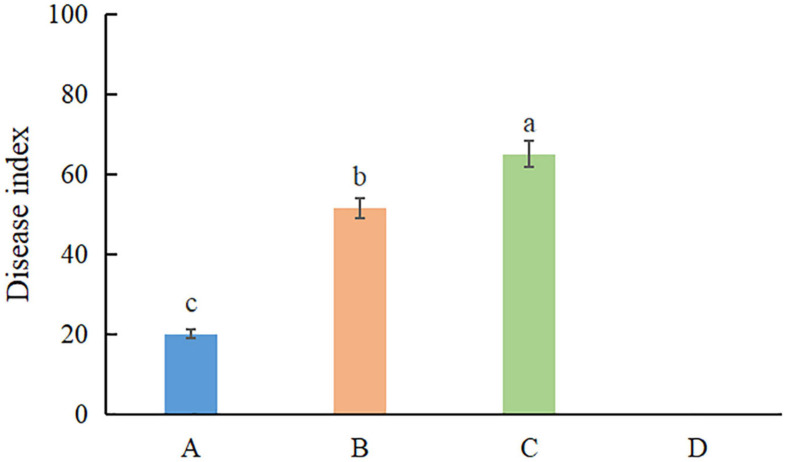
The disease index of inoculation of *Fusarium* species on *Alocasia modestum*. A, inoculation with *F. elaeidis* only. B, inoculation with *F. aglaonematis* only. C, inoculation with both *F. elaeidis* and *F. aglaonematis*. D, control, inoculation with sterilized water. a, b, and c indicate significant differences (*P* < 0.05).

**FIGURE 8:**
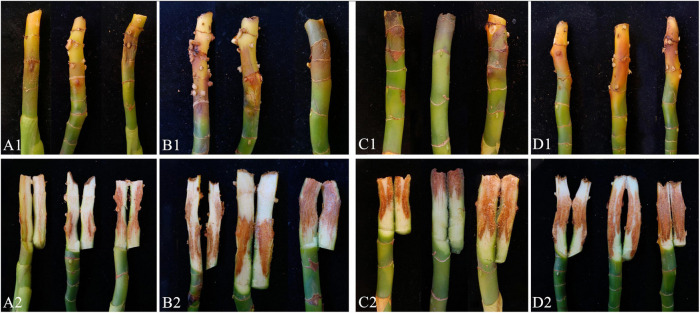
Inoculation test results of *Alocasia modestum* with and without physical injury. **(A1,A2)** Inoculation without physical injury using *F. aglaonematis* only. **(B1,B2)** Inoculation with physical injury using *F. aglaonematis* only. **(C1,C2)** Inoculation without physical injury using both *F. aglaonematis* and *F. elaeidis.* **(D1,D2)** Inoculation with physical injury using both *F. aglaonematis* and *F. elaeidis*.

Pathogenicity assays were conducted on potted *A. modestum* with either one fungal or both species. The results showed that these two *Fusarium* species could infect plants individually and a co-infection. All the inoculated plants showed stem rot, leaf wilted, and plants lodged (Figure 6), which were similar to the symptoms in the field. However, co-inoculation enhanced the disease severity. Moreover, *F. aglaonematis* alone was infected more severe than that *F. elaeidis.* Diseased plants inoculated with both species showed more severe rotted stems than single species and were easier to lodge (Figure 6). The disease index (DI) of inoculation with both species was 65, DI of single *F. aglaonematis* inoculation was 51, and DI of single *F. elaeidis* inoculation was 20. The disease index is given as a graph in Figure 7. Comparing the time of symptom appearance, plants inoculated with two species or single *F. aglaonematis* showed typical symptoms around 2 weeks after inoculation, earlier than that inoculated with single *F. elaeidis*, which showed typical symptoms usually 1 month after inoculation. Compared to the inoculation with physical injury and non-injury on the stems, both methods can develop typical symptoms on *A. modestum*, while inoculation with physical injury enhanced the disease severity, which showed more serious stem rots (Figure 8). Disease occurrence was higher in injured plants than in non-injured plants. In total, 12 plants were inoculated with a physical injury and 11 plants showed typical symptoms. Similar to that, all the 12 plants inoculated without an injury also developed symptoms. In the pathogenicity essays, when the pathogens were reisolated to fulfill Koch’s postulates, we observed a similar situation as the field condition, in which *F. elaeidis* was dominant and frequently isolated from co-infected plants. However, only *F. aglaonematis* was observed on co-inoculated rotted stems and developed fungal structures.

## Discussion

*Fusarium* is one of the most devastating pathogens on numerous agronomic, forestry, and horticultural crops worldwide (Wang et al., 2022). Therefore, correct species delineation is crucial for sustainable disease control of these pathogens (Jayawardena et al., 2021; Manawasinghe et al., 2021). Crous et al. (2021) re-examined the fusarioid taxa in *Nectriaceae* and showed that the Wollenweber concept of *Fusarium* presently encompasses 20 distinct genera. Moreover, *Fusarium* (=*Gibberella*) concept is delimited to 18 species complexes, including *Fusarium fujikuroi* species complex (FFSC) and the *Fusarium oxysporum* species complex (FOSC). Seven loci, namely, internal transcribed spacer (ITS), intergenic spacer (IGS), *tef1-*α, *cmdA*, *rpb1*, *rpb2*, and β*-tubulin*, were employed to study *Fusarium* and its allied genera from China (Wang et al., 2022); among these, the *rpb2* locus was most effective in species recognition in FFSC, followed by *tef1-*α, which is the most effective in the FOSC. However, ITS failed to resolve any species in *Fusarium* and IGS locus showed significant conflict with other loci on FOSC phylogenetic topology (Wang et al., 2022). However, the combined sequences of *cmdA*, *rpb1*, *rpb2*, *tef1-*α, and β*-tubulin* can significantly improve the species recognition of FFSC (Crous et al., 2021; Yilmaz et al., 2021; Wang et al., 2022). Lombard et al. (2019) on FOSC revealed that the *tef1-*α gene region and the *rpb2* gene region provided the best resolution to discriminate the novel species. In this study, we combined morphological characteristics with multiloci sequence analysis by applying *cmdA*, *rpb1*, *rpb2*, *tef1-*α, and β*-tubulin* for FOSC to ensure species identification accurately.

*Fusarium aglaonematis* was introduced as a new species while adding one more species to the *Fusarium fujikuroi* species complex (FFSC). Yilmaz et al. (2021) redefined species in FFSC and 67 species were accepted; later two novel species, *Fusarium prieskaense* and *Fusarium echinatum* were described by Crous et al. (2021) and another four novel species, namely, *Fusarium aquaticum*, *F. panlongense*, *Fusarium elaeagni*, and *Fusarium hechiense*, were described by Wang et al. (2022). Until now, 71 species have been included in FFSC (Wang et al., 2022). Previous studies showed that FFSC accommodates several biogeographically defined clades (O’Donnell et al., 1998). This includes two monophyletic Asian clades and an American clade and polyphyletic African clades (Crous et al., 2021; Yilmaz et al., 2021; Wang et al., 2022). Our new species clustered in Asian clades, in which the phylogenetic distribution is consistent with other species. *Fusarium elaeidis* from this study belongs to FOSC, which comprises 31 species (Lombard et al., 2019). Phylogenetically, our isolates from *A. modestum* clustered with *F. elaeidis* with high support. Morphologically, our isolates were similar to *F. elaeidis* described by Lombard et al. (2019).

Ten fungal species are associated with *Aglaonema modestum* (Farr and Rossman, 2022). Until now, there are no records of *Fusarium* species as a pathogen on *A. modestum.* However, *Fusarium* species have been reported from other *Aglaonema* species. *Fusarium subglutinans* has been reported to cause collar rot and the foliar blight on *Aglaonema commutatum*, which led to significant crop losses at commercial nurseries in Hawaii, United States (Uchida and Aragaki, 1994). Typical symptoms on *A. modestum* are similar to *A. commutatum*, while foliar blight was not observed on the former plants. *Fusarium elaeidis* has been reported as the pathogen of *Fusarium* wilt on oil palms and stem rot on *Alocasia longiloba* (Flood, 2006; Zhang et al., 2021). However, there are no records on *A. modestum.* This is the first report of *Fusarium* stem rot on *A. modestum* and co-infected by *F. aglaonematis* and *F. elaeidis.*

Pathogenicity tests showed that *Fusarium aglaonematis* and *F. elaeidis* have variations in the disease progression on *A. modestum. Fusarium aglaonematis* infects plants and develops symptoms more quickly than *F. elaeidis*, and the symptom caused by *Fusarium aglaonematis*, is more intense than that of *F. elaeidis*. While compared to a single infection of two species, co-infection of both species can enhance disease severity. Several studies have shown that co-infection can alter the disease responses in different host species. The co-infection of *Fusarium oxysporum* f. sp. *medicaginis* and *Rhizoctonia solani* in commercial alfalfa production has shown severe disease incidence, growth reduction, and biomass allocation across different varieties compared to a single infection (Fang et al., 2021). In addition, several studies have shown that being co-infected by different soil-borne pathogens has a significant increase in disease development compared with a single infection (Peters and Grau, 2002). Moreover, this could happen vice versa as co-infection might result in a more severe infection than a single infection (Vandemark et al., 2010; Lerch-Olson and Robertson, 2020). Thus, it is necessary to develop control measures focusing on co-infections rather than a single species (Barrett et al., 2009). Moreover, we observed that these two species can infect plants without a physical injury, yet wounds make plants more susceptible to being attacked by pathogens and developing more severe symptoms. Therefore, considering the importance of diseases, correct agricultural management measures should be taken, such as avoiding physical injuries to the plants by human activity and pests to control this disease.

## Conclusion

Overall in the present study, we isolated and characterized two *Fusarium* species causing stem rot in commercially grown *A. modestum* plants. These two species were identified as *F. elaeidis* and *F. aglaonematis*, a novel species. The pathogenicity of these two species was confirmed on potted *A. modestum* as single inoculations and co-inoculations. *Fusarium aglaonematis* is more pathogenic than *F. elaeidis*, while these two species infect together the disease severity is higher than a single infection. Moreover, physical injury can enhance the disease severity as well. This is the first study on co-infection by *Fusarium* species on *A. modestum*. Results from this study will enhance the knowledge of the *Fusarium* pathogenicity mechanism on commercially grown ornamental plants.

## Data Availability Statement

The datasets presented in this study can be found in online repositories. The names of the repository/repositories and accession number(s) can be found in the article/Supplementary Material.

## Author Contributions

YZ and ISM contributed to the experimental design. CC, ZM, and JL conducted the experiments. YZ, CC, and LN contributed to the data analysis. YZ, CY, and MX supplied experimental conditions. YZ prepared the manuscript. ISM, SSNM, and KDH revised the manuscript. All authors have contributed to the article and approved the submitted version of the manuscript.

## Conflict of Interest

The authors declare that the research was conducted in the absence of any commercial or financial relationships that could be construed as a potential conflict of interest.

## Publisher’s Note

All claims expressed in this article are solely those of the authors and do not necessarily represent those of their affiliated organizations, or those of the publisher, the editors and the reviewers. Any product that may be evaluated in this article, or claim that may be made by its manufacturer, is not guaranteed or endorsed by the publisher.

## Footnotes

1
http://www.iplant.cn/frps

2
https://powo.science.kew.org/

3
https://blast.ncbi.nlm.nih.gov/Blast.cgi

4
https://mafft.cbrc.jp/alignment/server/

5
http://www.phylo.org

6
http://www.indexfungorum.org
