# Supplementary material for: Co-infection of Fusarium aglaonematis sp. nov. and Fusarium elaeidis Causing Stem Rot in Aglaonema modestum in China
Source: Front Microbiol. 2022 Jun 30;13:930790. doi: 10.3389/fmicb.2022.930790 (PMC9279562; doi:10.3389/fmicb.2022.930790)
Supplement: Supplementary file 1 [file Table_1.pdf]

Supplementary Material

Supplementary Table 1 Strains of *Fusarium fujikuroi* species complex used in phylogenetic analysis

Species	Culture collection number	GenBank accession number				
		tef1	*tub2*	*cmdA*	*rpb2*	*rpb1*
F. acutatum	CBS 401.97	MW402124	MW402322	MW402458	MW402813	MW402652
	CBS 402.97[T]	MW402125	MW402323	MW402459	MW402768	MW402653
	CBS 739.97	AF160276	MW402348	AF158329	MN193883	MW402696
F. agapanthi	CBS 100193	MW401959	MW402160	MW402363	MW402727	MW402491
	NRRL 54463[T]	KU900630	KU900635	KU900611	KU900625	KU900620
	NRRL 54464	MN193856	KU900637	KU900613	KU900627	MW402718
F. aglaonemae	**ZHKUCC 22-0077**	**ON330437**	**ON330440**	**ON330434**	**ON330443**	**ON330446**
	**ZHKUCC 22-0078**	**ON330438**	**ON330441**	**ON330435**	**ON330444**	**ON330447**
	**ZHKUCC 22-0079**	**ON330439**	**ON330442**	**ON330436**	**ON330445**	**ON330448**
F. ananatum	CBS 118516[T]	LT996091	MN534089	MW402376	LT996137	MW402507
	CBS 118517	MN533988	MN534090	MN534157	MN534229	MW402508
	CBS 184.29	MW402105	MW402303	MW402445	MW402809	MW402629
F. andiyazi	CBS 119856	MN533989	MN534081	MN534174	MN534286	MW402523
	CBS 119857[T]	MN193854	LT996113	MN534175	LT996138	MW402524
F. annulatum	CBS 115.97	MW401973	MW402173	MW402373	MW402785	MW402503
	CBS 258.54[T]	MT010994	MT011041	MT010908	MT010983	MT010944
	CBS 135.95	MW402043	MW402242	MW402408	MW402745	MW402571
F. anthophilum	CBS 119858	MN533990	MN534091	MN534158	MN534232	MW402525
	CBS 119859	MN533991	MN534092	MN534164	MN534233	MW402526
	CBS 222.76[ET]	MW402114	MW402312	MW402451	MW402811	MW402641
F. aquaticum	LC13615	MW580446	MW533728	MW566273	MW474392	MW024437
	LC13616	MW580447	MW533729	MW566274	MW474393	MW024438
	LC7502 [T]	MW580448	MW533730	MW566275	MW474394	MW024439
F. awaxy	CBS 119831	MN534056	MN534108	MN534167	MN534237	MW402514
	CBS 119832	MN534057	MN534106	MN534170	MN534240	MW402515
F. bactridioides	CBS 100057[T]	MN533993	MN534112	MN534173	MN534235	MW402490
	NRRL 20476	AF160290	U34434	AF158343	–	–

Supplementary Table 1 (Continued)

Species	Culture collection number	GenBank accession number				
		tef1	*tub2*	*cmdA*	*rpb2*	*rpb1*
F. begoniae	CBS 403.97	MN193858	U61543	MW402460	MN193886	MW402654
	CBS 452.97[T]	MN533994	MN534101	MN534163	MN534243	MW402675
F. brevicatenulatum	CBS 404.97[T]	MN533995	MN534063	–	MN534295	MW402655
	CBS 100196	MN193859	–	–	MN193887	MW402492
F. bulbicola	CBS 220.76[T]	KF466415	KF466437	MW402450	MW402767	–
F. chinhoyiense	NRRL 25221[T]	MN534050	MN534082	MN534196	MN534262	MW402711
	NY 001B5	MN534051	MN534083	MN534197	MN534263	MW402725
F. circinatum	CBS 405.97[T]	MN533997	MN534097	MN534199	MN534252	MW402656
	CBS 141671	MW402083	MW402282	MW402427	MW402807	MW402610
	CBS 119864	MW401996	MW402196	MW402389	MW402736	MW402528
F. coicis	NRRL 66233[T]	KP083251	LT996115	LT996178	KP083274	–
F. concentricum	CBS 450.97[T]	AF160282	MW402334	MW402467	JF741086	MW402674
	CBS 453.97	MN533998	MN534123	MN534216	MN534264	MW402676
F. denticulatum	CBS 406.97	MN533999	MN534067	MN534185	MN534273	MW402657
	CBS 407.97[T]	MN534000	MN534068	MN534186	MN534274	MW402658
	CBS 735.97	AF160269	U61550	AF158322	LT996143	–
F. dlaminii	CBS 671.94	MN534004	MN534136	MN534152	MN534254	MW402690
	CBS 672.94	MN534005	MN534137	MN534153	MN534255	MW402691
	CBS 119860[T]	MW401995	MW402195	MW402388	KU171701	KU171681
F. echinatum	CBS 146496	MW834272	MW834300	MW834109	MW834003	MW834186
	CBS 146497[T]	MW834273	MW834301	MW834110	MW834004	MW834187
F. elaeagni	LC13627[T]	MW580466	MW533748	MW566293	MW474412	MW024457
	LC13628	MW580467	MW533749	MW566294	MW474413	MW024458
	LC13629	MW580468	MW533750	MW566295	MW474414	MW024459
F. ficicrescens	CBS 125177	MN534006	MN534071	MN534176	MN534281	MW402545
	CBS 125178[T]	KU604452	KP662896	KU603958	KT154002	MW402546
	CBS 125181	MN534007	MN534072	MN534177	MN534282	MW402548
F. fractiflexum	NRRL 28852[T]	AF160288	AF160315	AF158341	LT575064	–
F. fredkrugeri	CBS 408.97	MW402126	MW402324	MW402461	MW402814	–
	CBS 144209[T]	LT996097	LT996118	LT996181	LT996147	LT996199
	CBS 144495	LT996096	LT996117	LT996180	LT996146	LT996198

Supplementary Table 1 (Continued)

Species	Culture collection number	GenBank accession number				
		tef1	*tub2*	*cmdA*	*rpb2*	*rpb1*
F. fujikuroi	CBS 221.76^{T}	MN534010	MN534130	–	KU604255	MW402640
	CBS 257.52	MW402119	MW402317	MW402454	MW402812	MW402645
	CBS 265.54	MN534011	MN534132	MN534222	MN534268	MW402650
F. globosum	CBS 428.97^{T}	KF466417	MN534124	MN534218	KF466406	MW402668
	CBS 430.97	MN534013	MN534125	MN534219	MN534265	–
F. globosum (cont.)	CBS 120992	MW401998	MW402198	MW402390	MW402788	MW402529
F. guttiforme	CBS 409.97^{T}	MT010999	MT011048	MT010901	MT010967	MT010938
	NRRL 22945	AF160297	U34420	AF158350	JX171618	JX171505
F. hechiense	LC13644^{T}	MW580494	MW533773	MW566321	MW474440	MW024482
	LC13645	MW580495	MW533774	MW566322	MW474441	MW024483
	LC13646	MW580496	MW533775	MW566323	MW474442	MW024484
F. konzum	CBS 119849^{T}	LT996098	MN534095	LT996182	MW402733	MW402519
	CBS 139382	MW402071	MW402270	MW402418	MW402804	MW402598
	CBS 139383	MN534014	MN534094	MN534200	MN534244	MW402599
F. lactis	CBS 411.97^{ET}	MN193862	MN534077	MN534178	MN534275	MW402659
	CBS 420.97	MN534015	MN534078	MN534181	MN534296	MW402667
F. longicornicola	NRRL 52706^{T}	JF740788	MW402360	MW402487	JF741114	–
	NRRL 52712	JF740794	MW402361	MW402488	JF741120	MW402716
	NRRL 52713	JF740795	MW402362	MW402489	JF741121	MW402717
F. lumajangense	InaCCF872^{T}	LS479441	LS479433	–	LS479850	–
	InaCCF993	LS479442	LS479434	–	LS479851	–
F. madaense	CBS 146651	MW402096	MW402295	MW402437	MW402762	MW402617
	CBS 146656	MW402097	MW402296	MW402438	MW402763	MW402618
	CBS 146669^{T}	MW402098	MW402297	MW402439	MW402764	MW402619
F. mangiferae	CBS 119853	MN534016	MN534140	MN534225	MN534270	MW402522
	CBS 120994^{T}	MN534017	MN534128	MN534224	MN534271	MW402530
	NRRL 25226	AF160281	U61561	AF158334	HM068353	MW402712
F. mexicanum	NRRL 47473	GU737416	GU737308	GU737389	LR792615	LR792579
	NRRL 53147^{T}	GU737282	GU737494	–	MN724973	MG838088
F. mundagurra	RGB5717T	KP083256	MN534146	MN534214	KP083276	–
F. musae	CBS 624.87^{T}	FN552086	FN545368	MW402474	MW402772	MW402689
	NRRL 28893	FN552092	FN545374	FN552070	FN552114	–

Supplementary Table 1 (Continued)

Species	Culture collection number	GenBank accession number				
		tef1	*tub2*	*cmdA*	*rpb2*	*rpb1*
F. napiforme	CBS 748.97^T	MN193863	MN534085	MN534192	MN534291	MW402701
	CBS 135139	MN534019	MN534084	MN534183	MN534290	MW402572
	CBS 135141	MW402045	MW402244	–	MW402797	MW402573
F. nirenbergiae	CBS 744.97	AF160312	U34424	AF158365	LT575065	–
F. nygamai	CBS 749.97^T	MW402151	MW402352	MW402479	EF470114	MW402703
	CBS 834.85	MW402154	MW402355	MW402482	MW402821	MW402707
	CBS 119852	MW401992	MW402192	MW402386	MW402734	MW402521
F. ophioides	CBS 118512^T	MN534022	MN534118	MN534209	MN534303	–
	CBS 118513	MN534023	MN534119	MN534202	MN534300	–
	CBS 118514	MN534024	MN534117	MN534206	MN534302	–
F. panlongense	LC13656^T	MW580510	MW533789	MW566337	MW474456	MW024498
	MUCL 55958	LT574913	LT575078	LT575159	LT574994	–
	MUCL 55950	LT574905	LT575070	LT575151	LT574986	–
	MUCL 55954	LT574909	LT575074	LT575155	LT574990	–
F. parvisorum	CMW 25267^T	KJ541060	KJ541055	–	–	–
F. phyllophilum	CBS 216.76^T	MN193864	KF466443	KF466333	KF466410	MW402637
	CBS 246.61	MW402118	MW402316	MW402453	–	MW402644
F. pilosicola	NRRL 29123	MN534054	MN534098	MN534165	MN534247	–
	NRRL 29124^T	MN534055	MN534099	MN534159	MN534248	–
F. prieskaense	CPC 30825	MW834274	MW834302	MW834111	MW834006	MW834189
	CBS 146498^T	MW834275	MW834303	MW834112	MW834007	MW834190
	CBS 146499	MW834276	MW834304	MW834113	MW834008	MW834191
F. proliferatum	CBS 480.96E^T	MN534059	MN534129	MN534217	MN534272	–
F. pseudoanthophilum	CBS 414.97^T	MW402128	MW402326	MW402463	–	MW402661
	CBS 745.97	MW402148	MW402349	MW402476	MW402820	MW402697
F. pseudocircinatum	CBS 449.97^T	AF160271	MN534069	MN534190	MN534277	MW402673
	CBS 455.97	MN534029	MN534070	MN534184	MN534276	–
F. pseudonygamai	CBS 416.97	MN534030	MN534064	MN534194	MN534283	MW402663
	CBS 417.97^T	AF160263	MN534066	AF158316	MN534285	MW402664
	CBS 484.94	MN534031	MN534065	MN534195	MN534284	MW402681
F. ramigenum	CBS 418.97^T	KF466423	MN534145	MN534187	KF466412	MW402665

Supplementary Table 1 (Continued)

Species	Culture collection number	GenBank accession number				
		tef1	*tub2*	*cmdA*	*rpb2*	*rpb1*
F. ramigenum	CBS 526.97	MN534032	MN534086	MN534188	MN534292	MW402682
F. sacchari	CBS 223.76[ET]	MW402115	MW402313	AF158331	JX171580	–
	CBS 131372	MN534033	MN534134	MN534226	MN534293	MW402560
	CBS 131370	MW402031	MW402230	MW402404	MW402793	MW402558
F. secorum	NRRL 62593[T]	KJ189225	–	KJ189235	–	–
	NRRL 62594	KJ189228	–	KJ189238	–	–
F. siculi	CBS 142222[T]	LT746214	LT746346	LT746189	LT746327	–
	CPC 27189	LT746215	LT746347	LT746190	LT746328	–
F. sterilihyposum	NRRL 25623[T]	MN193869	AF160316	AF158353	MN193897	MW402713
F. Sterilihyposum (cont.)	NRRL 53991	GU737413	GU737305	GU737386	–	–
	NRRL 53997	GU737414	GU737306	GU737387	–	–
F. subglutinans	CBS 536.95	MW402139	MW402339	MW402471	MW402836	MW402685
	CBS 747.97[NT]	MW402150	MW402351	MW402478	MW402773	MW402700
	CBS 136481	MW402059	MW402258	MW402413	MW402748	MW402585
F. succisae	CBS 187.34	MW402109	MW402307	MW402448	MW402810	–
	CBS 219.76[ET]	AF160291	U34419	AF158344	MW402766	MW402639
F. sudanense	CBS 454.97[T]	MN534037	MN534073	MN534179	MN534278	MW402677
	CBS 675.94	MN534038	MN534074	MN534182	MN534279	MW402693
F. temperatum	CBS 135538	MN534039	MN534111	MN534168	MN534239	MW402575
	CBS 135539	MN534040	MN534110	MN534169	MN534242	MW402576
	MUCL 52463[T]	–	MW402359	MW402486	MW402776	–
F. terricola	CBS 483.94[T]	MN534042	MN534076	MN534189	LT996156	MW402680
	CBS 119850	MN534041	MN534075	MN534180	MN534280	MW402520
F. thapsinum	CBS 776.96[T]	MN534044	MN534080	–	MN534289	MW402704
	CBS 100312	MW401961	MW402162	MW402365	MW402780	MW402494
	CBS 100313	MW401962	MW402163	MW402366	MW402781	MW402495
F. tjaetaba	NRRL 66243[T]	KP083263	GU737296	LT996187	KP083275	–
F. tupiense	CML345	DQ452861	DQ445783	–	–	–
F. udum	CBS 178.32	AF160275	U34433	MW402442	LT996172	MW402624
	CBS 747.79	MN193872	MN534141	MN534154	MN534258	MW402699
F. verticillioides	CBS 125.73	MW402012	MW402212	MW402392	MW402791	MW402543
F. verticillioides	CBS 447.95	MW402133	MW402332	MW402466	MW402770	MW402671

Supplementary Table 1 (Continued)

Species	Culture collection number	GenBank accession number				
		tef1	*tub2*	*cmdA*	*rpb2*	*rpb1*
F. verticillioides	CBS 531.95	MW402136	MW402336	MW402468	MW402771	MW402683
F. volatile	CBS 143874[T]	LR596007	LR596008	MK984595	LR596006	–
F. volatile	NRRL 25615	AF160304	AF160320	AF158357	–	–
F. werrikimbe	CBS 125535[T]	–	MN534104	MN534203	MN534304	–
F. xylarioides	CBS 258.52[T]	MN193874	AY707118	MW402455	HM068355	MW402646
F. xylarioides	CBS 749.79	MN534049	MN534143	AF158326	MN534259	MW402702
F. xyrophilum	NRRL 62710	MN193875	–	–	MN193903	MW402720
	NRRL 62721[T]	MN193877	–	–	MN193905	MW402721

The isolates obtained in this study are bold.
T=Ex-type specimen. ET=Ex-epitype specimen.
