# Supplementary material for: Co-infection of Fusarium aglaonematis sp. nov. and Fusarium elaeidis Causing Stem Rot in Aglaonema modestum in China
Source: Front Microbiol. 2022 Jun 30;13:930790. doi: 10.3389/fmicb.2022.930790 (PMC9279562; doi:10.3389/fmicb.2022.930790)
Supplement: Supplementary file 2 [file Table_2.pdf]

Supplementary Material

Supplementary Table 2 Strains of *Fusarium oxysporum* species complex used in phylogenetic analysis

Species name	Culture collection number	GenBank accession number		
		rpb2	*tef1*	*tub2*
Fusarium callistephi	CBS 187.53[T]	MH484875	MH484966	MH485057
	CBS 115423	MH484905	MH484996	MH485087
F.carminascens	CBS 144739	MH484934	MH485025	MH485116
	CBS 144740	MH484935	MH485026	MH485117
	CBS 144741	MH484936	MH485027	MH485118
F.contaminatum	CBS 111552	MH484900	MH484991	MH485082
	CBS 114899[T]	MH484901	MH484992	MH485083
	CBS 117461	MH484911	MH485002	MH485093
F.cugenangense	CBS 620.72	MH484879	MH484970	MH485061
	CBS 130304	MH484921	MH485012	MH485103
	CBS 130308	MH484920	MH485011	MH485102
F.curvatum	CBS 247.61	MH484876	MH484967	MH485058
	CBS 238.94[T]	MH484893	MH484984	MH485075
	CBS 141.95	MH484894	MH484985	MH485076
F.duoseptatum	CBS 102026	MH484896	MH484987	MH485078
F.elaeidis	CBS 217.49	MH484870	MH484961	MH485052
	CBS 218.49	MH484871	MH484962	MH485053
	CBS 255.52	MH484874	MH484965	MH485056
	**ZHKUCC 22-0080**	**ON330431**	**ON330425**	**ON330428**
	**ZHKUCC 22-0081**	**ON330432**	**ON330426**	**ON330429**
	**ZHKUCC 22-0082**	**ON330433**	**ON330427**	**ON330430**
F.fabacearum	CBS 144742	MH484938	MH485029	MH485120
	CBS 144743	MH484939	MH485030	MH485121
	CBS 144744	MH484940	MH485031	MH485122
F.foetens	CBS 120665	MH484918	MH485009	MH485100
F.glycines	CBS 176.33	MH484868	MH484959	MH485050
	CBS 214.49	MH484869	MH484960	MH485051
	CBS 200.89	MH484888	MH484979	MH485070
F.gossypinum	CBS 116611	MH484907	MH484998	MH485089
	CBS 116612	MH484908	MH484999	MH485090
	CBS 116613[T]	MH484909	MH485000	MH485091
F.hoodiae	CBS 132474[T]	MH484929	MH485020	MH485111
	CBS 132476	MH484930	MH485021	MH485112
	CBS 132477	MH484931	MH485022	MH485113
F.languescens	CBS 645.78[T]	MH484880	MH484971	MH485062
	CBS 646.78	MH484881	MH484972	MH485063
	CBS 413.9	MH484890	MH484981	MH485072
F.libertatis	CBS 144748	MH484932	MH485023	MH485114
	CBS 144747	MH484933	MH485024	MH485115
	CBS 144749[T]	MH484944	MH485035	MH485126

F.nirenbergiae	CBS 129.24	MH484864	MH484955	MH485046
	CBS 149.25	MH484865	MH484956	MH485047

Supplementary Table 2 (Continued)

Species name	Culture collection number	GenBank accession number		
		rpb2	*tef1*	*tub2*
F.nirenbergiae	CBS 181.32	MH484867	MH484958	MH485049
	CBS 758.68	MH484877	MH484968	MH485059
	CBS 744.79	MH484882	MH484973	MH485064
	CBS 127.81	MH484883	MH484974	MH485065
F.odoratissimum	CBS 794.7	MH484878	MH484969	MH485060
	CBS 102030	MH484898	MH484989	MH485080
	CBS 130310	MH484922	MH485013	MH485104
F.oxysporum	CBS 221.49	MH484872	MH484963	MH485054
	CBS 144134^{ET}	MH484953	MH485044	MH485135
	CBS 144135	MH484954	MH485045	MH485136
	CPC 25822	MH484943	MH485034	MH485125
F.pharetrum	CBS 144750	MH484951	MH485042	MH485133
	CBS 144751^{T}	MH484952	MH485043	MH485134
F.trachichlamydosporum	CBS 102028	MH484897	MH484988	MH485079
F.triseptatum	CBS 258.5^{T}	MH484873	MH484964	MH485055
	CBS 116619	MH484910	MH485001	MH485092
	CBS 119665	MH484916	MH485007	MH485098
F.udum	CBS 177.31	MH484866	MH484957	MH485048
F.veterinarium	CBS 109898^{T}	MH484899	MH484990	MH485081
	CBS 117787	MH484912	MH485003	MH485094
	CBS 117790	MH484913	MH485004	MH485095

The isolates obtained in this study are bold.
T=Ex-type specimen. ET=Ex-epitype specimen.
